# Tungstate Reduces the Expression of Gluconeogenic Enzymes in STZ Rats

**DOI:** 10.1371/journal.pone.0042305

**Published:** 2012-08-08

**Authors:** Laura Nocito, Delia Zafra, Joaquim Calbó, Jorge Domínguez, Joan J. Guinovart

**Affiliations:** 1 Institute for Research in Biomedicine (IRB Barcelona), Barcelona, Spain; 2 Centro de Investigación Biomédica en Red de Diabetes y Enfermedades Metabólicas Asociadas (CIBERDEM), Madrid, Spain; 3 Department of Biochemistry and Molecular Biology, University of Barcelona, Barcelona, Spain; Universidade de Brasília, Brazil

## Abstract

**Aims:**

Oral administration of sodium tungstate has shown hyperglycemia-reducing activity in several animal models of diabetes. We present new insights into the mechanism of action of tungstate.

**Methods:**

We studied protein expression and phosphorylation in the liver of STZ rats, a type I diabetes model, treated with sodium tungstate in the drinking water (2 mg/ml) and in primary cultured-hepatocytes, through Western blot and Real Time PCR analysis.

**Results:**

Tungstate treatment reduces the expression of gluconeogenic enzymes (PEPCK, G6Pase, and FBPase) and also regulates transcription factors accountable for the control of hepatic metabolism (c-jun, c-fos and PGC1α). Moreover, ERK, p90rsk and GSK3, upstream kinases regulating the expression of c-jun and c-fos, are phosphorylated in response to tungstate. Interestingly, PKB/Akt phosphorylation is not altered by the treatment. Several of these observations were reproduced in isolated rat hepatocytes cultured in the absence of insulin, thereby indicating that those effects of tungstate are insulin-independent.

**Conclusions:**

Here we show that treatment with tungstate restores the phosphorylation state of various signaling proteins and changes the expression pattern of metabolic enzymes.

## Introduction

Sodium tungstate is an effective anti-diabetic agent. Its main advantages are its low toxicity profile and the fact that it is active when administered orally. Oral administration of sodium tungstate to streptozotocin (STZ) -induced diabetic rats causes a dramatic decrease in blood glucose concentration [1], [2], restores hepatic glucose metabolism, increases the total amount and translocation of GLUT4 in muscle [3], [4] and decreases the activity of sucrase and the entering of glucose into the jejunum through the SGLT1 Na^+^/D-Glucose cotransporter [5]. Tungstate also has anti-obesity properties when administered orally to diet-induced obese rats since this compound significantly decreases body weight gain and adiposity without modifying caloric intake or growth rate in these animals [6]. Moreover, it has been recently discovered that the control of food intake and body weight by tungstate is mediated by the activation of the hypothalamic leptin pathway [7].

Previous studies in our group demonstrated that tungstate increases glycogen deposition in primary cultured hepatocytes, thereby exerting insulin-like actions in these cells. Analysis of the effects of this compound on several components of the insulin signaling transduction cascade in cultured cells demonstrated that they are not mediated by the insulin receptor (IR) as its phosphorylation state remains unchanged after treatment [8]. In contrast, a clear transient phosphorylation of ERK (extracellular signal-regulated kinases 1 and 2) is detected. Tungstate also triggers the phosphorylation of p90rsk and glycogen synthase kinase-3β (GSK3β) and the activation of glycogen synthase (GS) in hepatocytes.

Here we studied the mechanism of action of tungstate *in vivo* on several pathways potentially implicated in the improvement of the diabetic state. For this purpose, in STZ-induced diabetic rats treated with tungstate, we analyzed changes in the phosphorylation and the expression of key enzymes and transcription factors involved in the regulation of gluconeogenesis, one of the most relevant processes altered in the liver of diabetic patients. Here we show that a 30-day treatment with this compound restores the phosphorylation state of various signaling proteins and changes the expression pattern of regulatory enzymes.

## Materials and Methods

### Ethics Statement

All procedures were approved by the Barcelona Science Park's Animal Experimentation Committee (CEEA-PCB, permit number P09-R4-07) and were carried out in accordance with the European Community Council Directive and the National Institute of Health guidelines for the care and use of laboratory animals.

### Chemicals

Sodium tungstate was from Carlo Erba (Milan, Italy). STZ was from Sigma-Aldrich (St. Louis, MO). Enzymes and biochemical reagents were from either HORIBA ABX or Sigma-Aldrich. All other chemicals were of analytical grade. ERK antibody was from Upstate (Waltham, MA). GSK3α/β antibody was from Santa Cruz Biotechnology (Santa Cruz, CA). PKB/Akt antibody was from Sigma-Aldrich. Phospho-GSK3α/β (Ser 21/9), phospho-ERK (Thr 202/Tyr 204), phospho-p90rsk (Ser 380) and p90rsk antibodies were from Cell Signalling (Beverly, MA). Phospho-PKB/Akt (Ser 473) antibody was from NanoTools (Teningen, Germany). GAPDH antibody was from Ambion (Austin, USA). PEPCK antibody was a kind gift of Dr. Daryl Granner (Vanderbilt University, USA).

### Animals

Adult male Wistar rats (200 g) were kept under a constant 12-h light-dark cycle and were allowed to eat and drink *ad libitum*. When stated, diabetes was induced by a single intravenous injection of STZ (60 mg/kg of body weight) in 0.9% NaCl with 100 mM sodium citrate buffer (pH 4.5). Diabetes was confirmed by determination of glycemia (glucose meter and glycemia strips, Bayer). Diabetic rats were used 5–7 days after STZ injection. At the beginning of the experiment, the animals were divided into four groups: control (Ctrol), diabetic (Diab), control treated with tungstate (Ctrol+W) and diabetic treated with tungstate (Diab+W). The non-treated animals were given normal drinking water while the treated ones received a solution of 2 mg/ml sodium tungstate in distilled water. The treatment was carried out for 30 days. During this period, glycemia, fluid and food intake were measured every three days between 10 and 12 a.m. At the end of the experiment, rats were anesthetized with Isofluorane 2% (Isoba vet, Schering Plough) and heart blood was collected to measure serum parameters. Afterwards, organs were rapidly frozen in liquid nitrogen for later determinations. Animals were sacrificed between 10 and 12 a.m.

### Enzyme Activity and Metabolite Determination

GS activity was determined in frozen liver samples homogenized (Polytron) in 10 volumes of ice-cold 10 mM Tris-HCl buffer (pH 7.4) containing 150 mM KF, 15 mM EDTA, 0.6 M sucrose, 1 mM phenylmethylsulfonyl fluoride, 1 mM benzamidine, 10 µg/ml leupeptin, 10 µg/ml pepstatin, 10 µg/ml aprotinin, 25 nM okadaic acid and 15 mM 2-mercaptoethanol. Whole homogenates were used for determinations. Total (T) GS activity (measured in the presence of 6.6 mM G6P), intrinsic (I) GS activity (measured in the absence of G6P) and GS I/T activity ratio (−G6P/+G6P) were measured as described [9]. Protein concentration was measured following the method Pierce BCA (bicinchoninic acid) protein assay. Frozen liver samples were homogenized with 4 volumes of 30% (w/v) KOH and boiled at 100°C for 15 min; glycogen was determined after ethanol precipitation as described [10].

### Electrophoresis and Immunoblotting

Homogenates (20 µg of protein) were resolved by 10% SDS-PAGE. The protein was transferred onto a nitrocellulose membrane and probed with the mentioned primary antibodies. Secondary antibodies conjugated to horseradish peroxidase against rabbit (GEHealthcare), mouse (DakoCytomation) or sheep (DakoCytomation) immunoglobulins were used. Immunoreactive bands were visualized using an ECLplus kit (GE Healthcare), following the manufacturer's instructions. Densitometric analyses were carried out using NIH Image J software.

### Serum Parameters

Blood glucose was measured using an Ascensia Brio Glucose Analyzer (Bayer). Serum glucose (HORIBA ABX) and triglyceride (Sigma-Aldrich) concentrations were measured spectrophotometrically by standard techniques adapted to a COBAS Mira analyzer (Roche Diagnostics). Serum insulin levels were measured by immunoassay (Spi Bio).

### Hepatocyte isolation and culture

Collagenase perfusion was used to isolate hepatocytes from male Wistar rats (180–225 g) previously fasted for 24 h, as described [11]. Hepatocytes were suspended in DMEM supplemented with 10 mM glucose, 10% FCS, 100 nM insulin and 100 nM dexamethasone, seeded onto 60-mm diameter plastic plates and processed as described [8]. After 4 h, medium was replaced by serum-free DMEM and cells were left overnight. Specific treatments were then performed, depending on the experiments. For the determination of gene expression changes under gluconeogenic conditions, medium was replaced by glucose-free and FBS-free DMEM, and after 3 h cells were treated with dexamethasone 100 nM (Dex), sodium tungstate 1 mM (W) or both (Dex+W) for 18 h.

### Glucose production assay

Primary hepatocytes were cultured in p60 plates (1.74×10^6^ cells per plate) following the hepatocyte isolation and culture method described in the previous paragraph. After incubating the cells overnight in serum- and glucose-free DMEM, the medium was replaced with 3 ml of glucose production buffer consisting of glucose-free DMEM supplemented with 20 mM sodium lactate and 2 mM sodium pyruvate [12]. After a 3-h incubation, 0.5 ml of medium was collected and the glucose concentration was measured spectrophotometrically by standard techniques (HORIBA ABX) adapted to a COBAS Mira analyzer (Roche Diagnostics).The readings were then normalized to the total protein content determined from the whole-cell lysates.

### RNA Purification and Retrotranscription

Total RNA was isolated from rat liver tissue by homogenizing (Polytron) 100 mg of sample in 1 ml of TRIzol (Invitrogen). After centrifugation at 12,000×*g* for 5 min, 0.2 ml of chloroform was added to the supernatant and it was then centrifuged again at 12,000×*g* for 15 min at 4°C to obtain two phases. Total RNA was then precipitated by adding 0.5 ml of isopropyl alcohol to the aqueous phase. After an incubation of 10 min at room temperature, samples were centrifuged at 12,000×*g* for 10 min at 4°C. Pellets were washed with 1 ml of 70% ethanol and centrifuged at 7500×*g* for 5 min at 4°C. The desiccated pellets were resuspended in 100 µl of RNase-free water. 5 µg of total RNA from each sample was reverse-transcribed for 50 min at 42°C in a 20-µl reaction volume using SuperScript III reverse transcriptase, following the manufacturer's instructions (SuperScript First-strand Synthesis System for RT-PCR, Invitrogen), in the presence of 50 ng of random hexamers.

### Quantitative Real-time PCR

PCR tests were performed at the Genomic Unit of Core Scientific Services at the University of Barcelona following the standard Real-time PCR protocol of the ABI Prism 7900 Detection System, together with the appropriate ready-made TaqMan primer/probe sets (Applied Biosystems). Each sample was analyzed from triplicate wells. The temperature profile consisted of 40 cycles of 15 s at 95°C and 1 min at 60°C. Data were analyzed with the 2^ΔΔ*Ct*^ method using 18S rRNA as endogenous reference.

### Statistical Analysis

Data are expressed as the mean ± S.E.M. Statistical significance was determined by unpaired Student's *t* test using Microsoft Excel (version XP; Microsoft Corp., Redmond, WA). Statistical significance was assumed at *P*≤0.05.

## Results

### Tungstate treatment ameliorates hyperglycemia and normalizes the expression of key regulatory enzymes in STZ diabetic rats

Healthy and STZ-diabetic rats were treated for 30 days with oral sodium tungstate (2 mg/ml) dissolved in distilled water. Non-treated animals of each group were given tap water. We observed a slight decrease in water intake in healthy treated animals compared to the non-treated ones. In the case of diabetic treated animals, there was a drastic decrease in water intake compared to their controls (Figure S1). As previously reported, blood glucose concentration was dramatically decreased in treated diabetic animals compared to untreated diabetic ones. The opposite pattern was observed for glycogen content and for the GS I/T (−G6P/+G6P) activity ratio, an estimate of the degree of GS activation (Table 1). We also observed a tungstate-mediated moderate increment in the levels of insulin in the blood of STZ-diabetic rats. Tungstate treatment of healthy rats did not cause significant changes in glycemia, serum insulin concentration and GS total activity, but it reduced the liver glycogen content. Serum triglyceride, a parameter that was increased in STZ-diabetic rats, was also lowered after tungstate treatment in both healthy and STZ-diabetic rats (Table 1).

**Table 1:** Effects of tungstate on glycemia and insulinemia, and on glycogen content and glycogen synthase activity in liver.

Metabolic measurements				
	Ctrol (6)	Ctrol+W (7)	Diab (9)	Diab+W (14)
**Glycaemia (mg/dl)**	127.5±4.09	120.7±5.10	575.56±13.33***	261.71±23.94†††
**Glycogen (mg/g liver)**	36.58±2.03	25.35±3.37*	14.77±1.83***	29.29±1.04†††
**GS Total Activity (mU/mg)**	1.94±0.13	1.96±0.14	2.16±0.10	1.88±0.11
**GS I Activity (mU/mg)**	0.27±0.026	0.22±0.008	0.16±0.011**	0.18±0.010*
**I/T Ratio**	0.14±0.005	0.11±0.008	0.07±0.004***	0.10±0.005†††
**Triglycerides (mmol/l)**	1.79±0.15	0.98±0.21**	2.86±0.41*	0.86±0.07††
**Serum insulin (ng/ml)**	1.93±0.36	1.59±0.40	0.24±0.05*	0.99±0.18†††

Figures in brackets indicate the number of animals used to calculate the mean for each condition. Values are given as mean ± S.E.M.

* *, P<0.05;*

** *, P<0.01;*

*** *, P<0.001* compared with control and

†† *, P<0.01;*

††† *, P<0.001* compared with diabetic animals.

**Figure 1:**
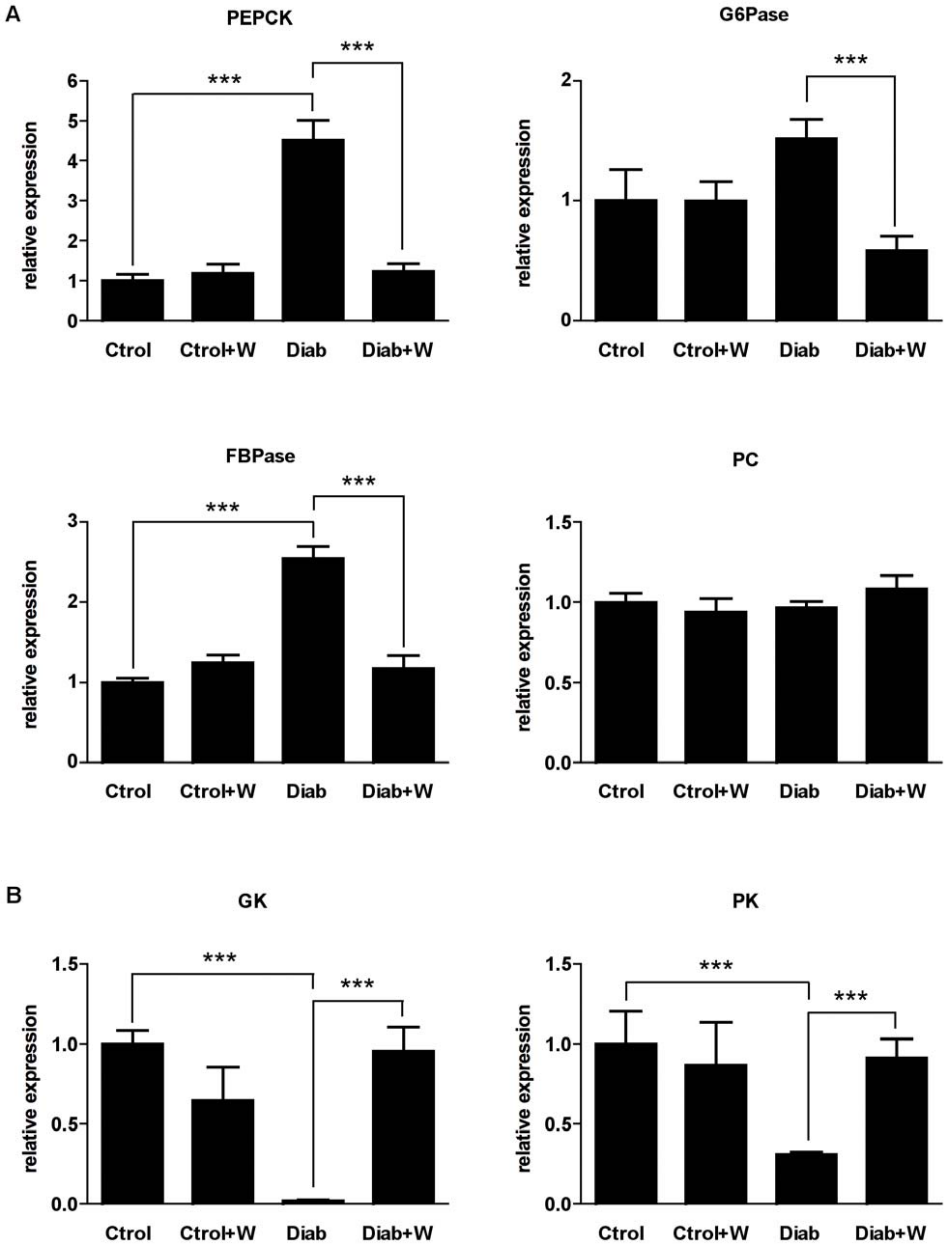
Tungstate-induced changes in the hepatic expression of metabolic genes. Total mRNA was extracted and Real time-PCR analysis was performed in the livers of all the experimental groups. Effects of the treatment on the expression of the gluconeogenic enzymes PEPCK, G6Pase, FBPase and PC (**A**) and glycolytic enzymes GK and PK (**B**). (n = 6 —14 in each group). Error bars represent S.E.M. ***, *P<0.001*.

**Figure 2:**
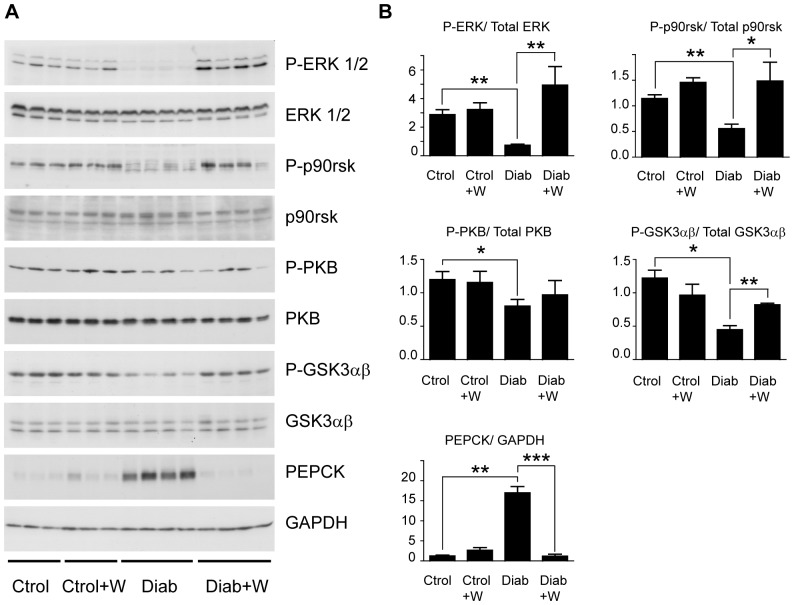
Tungstate-induced changes in protein phosphorylation. Phosphorylation of ERK, p90rsk, PKB and GSK3αβ was analyzed by Western blot using phospho-specific antibodies. Total ERK, total p90rsk, total PKB, total GSK3αβ and GAPDH were used as loading controls. Protein expression and phosphorylation were quantified by densitometry of the corresponding Western blot signal. **A** The images are representative of 3 controls (Ctrol), 3 controls treated with tungstate (Ctrol+W), 4 diabetic animals (Diab), and 4 diabetic animals treated with tungstate (Diab+W) from two independent experiments. In each experiment n = 6 for Ctrol, n = 7 for Ctrol+W, n = 9 for Diab and n = 14 for Diab+W. 20 µg of protein were loaded per well. Analysis of PEPCK expression in the same experimental groups serves as a control of tungstate action in those samples. **B** Bar graphs represent relative density (phospholylated versus total; PEPCK versus GAPDH) from Western blot signal (n = 3–8 in each group). Error bars represent S.E.M. *, P<0.05; **, P<0.01; ***, *P<0.001*.

A key effect of the 15-day treatment with tungstate in the STZ rat is a normalization of hepatic phosphoenolpyruvate carboxykinase (PEPCK) mRNA levels [1]. Here we examined whether this effect was also observed in a 30-day treatment (Figure 1A). Indeed, tungstate treatment reduced the expression of PEPCK to basal levels. This effect was accompanied by the normalization of two more transcriptionally-regulated gluconeogenic enzymes, namely glucose-6-phosphatase (G6Pase) and fructose-1,6-bisphosphatase (FBPase). In contrast, pyruvate carboxylase (PC) did not show any alteration at the mRNA level (Figure 1A). Remarkably, no effect was observed in treated healthy animals. Previous studies showed that the treatment of STZ rats with tungstate stimulated the expression of hepatic glucokinase (GK) and pyruvate kinase (L-PK), which were measured by Northern Blot [1], [2]. The normalizing effects on glycolytic gene expression were also observed in our 30-day experiments, as shown by RT-PCR (Figure 1B). Expression of PEPCK was also tested at protein levels. PEPCK was upregulated in STZ rats and normalized by tungstate treatment (Figure 2).

### Phosphorylation of ERK, GSK3 and p90rsk is impaired in livers of STZ diabetic rats and normalized after tungstate treatment

One of the hallmarks of tungstate is its capacity to increase ERK phosphorylation in several cell lines [4], [8], [13]. This enhanced phosphorylation has been considered responsible for the changes in glycogen content observed in primary cultured rat hepatocytes [8].

Using Western blot analysis with phospho-specific antibodies, we first observed that ERK phosphorylation is dramatically decreased in livers of STZ diabetic rats, at two (data not shown) and five weeks after STZ injection (Figure 2). However, ERK phosphorylation was recovered after tungstate treatment. In contrast, this parameter was not increased in treated healthy animals and total ERK expression was not affected in any of the conditions studied.

We also analyzed the phosphorylation state of several proteins of the insulin signaling cascade. In addition to ERK phosphorylation, p90rsk and GSK3 phosphorylation was also decreased in the livers of STZ-diabetic animals (Figure 2). Tungstate treatment also restored p90rsk and GSK3 phosphorylation in these rats. However, PKB phosphorylation was not modified by this treatment (Figure 2). In fact, a slight decrease in PKB phosphorylation was detected in untreated diabetic animals, but no significant recovery was observed in the treated ones.

### Effects of tungstate on the expression of key transcription factors

**Figure 3:**
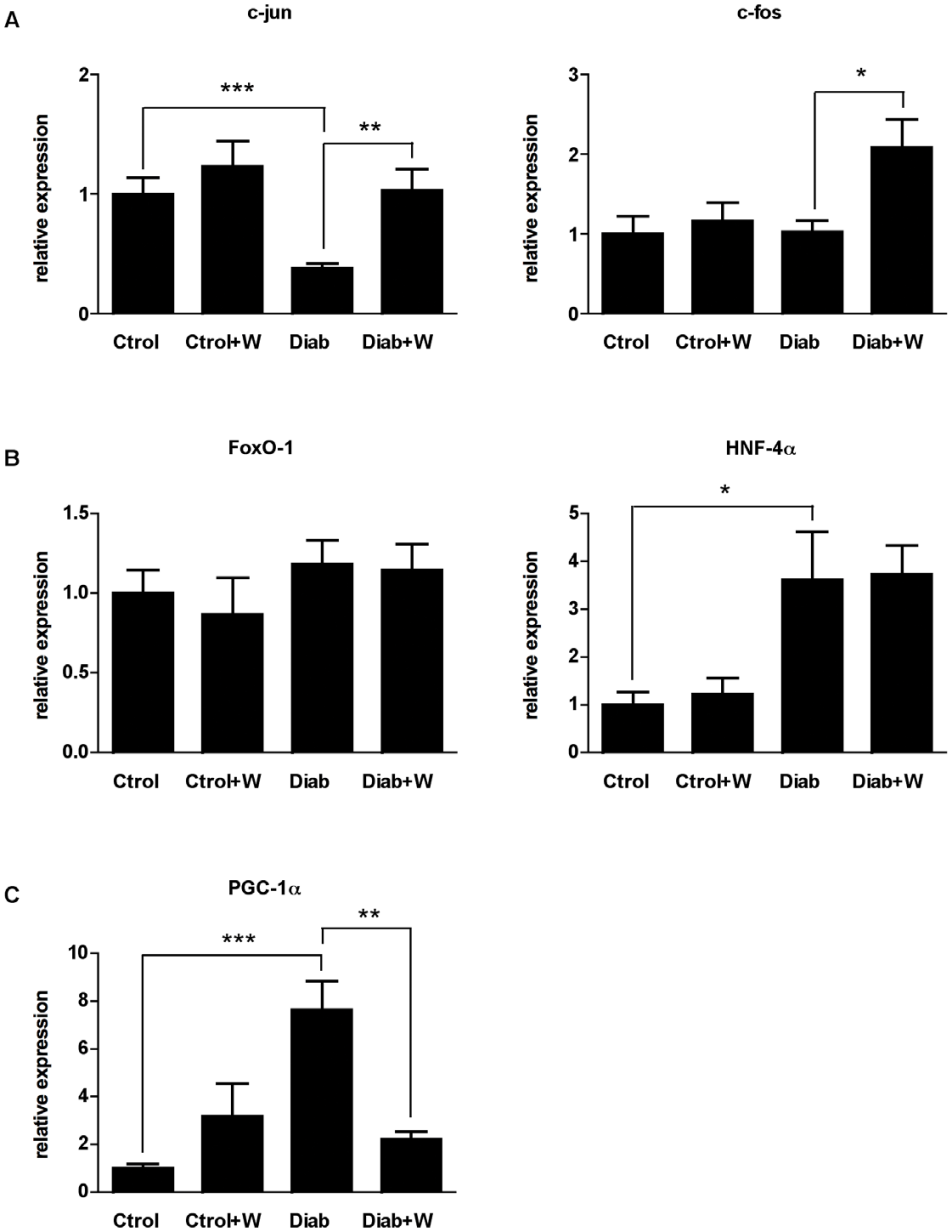
Tungstate-induced changes in the expression of transcription factors involved in metabolic control. Total mRNA was extracted and Real time-PCR analysis was performed in the livers of all the experimental groups. Effects of tungstate on c-jun and c-fos expression (**A**) and on FoxO-1, HNF-4α, and PGC-1α (**B**). (n = 6–14 in each group). Error bars represent S.E.M. *, P<0.05; **, P<0.01; ***, *P<0.001*.

In order to identify the mechanism of action by which tungstate decreases the expression of gluconeogenic enzymes, we analyzed whether this compound also modifies the expression of the main transcription factors and coactivators involved in the regulation of PEPCK and G6Pase, the rate-limiting enzymes of gluconeogenesis. The phosphorylation of ERK-1/2 and of GSK3 induced by tungstate in livers of STZ rats led us to focus first on the expression of c-jun and c-fos, which act downstream of ERK and GSK3 and are involved in the negative regulation of hepatic gluconeogenesis [14], [15], [16]. Treatment with tungstate restored hepatic c-jun mRNA in diabetic animals (Figure 3A). Tungstate also caused a net increase in c-fos expression in diabetic but not in healthy animals.

Next, we analyzed the expression of two other well-known transcriptional regulators of gluconeogenic enzymes, forkhead box 1 (FoxO-1) and hepatic nuclear factor-4 alpha (HNF-4α) [17], [18], [19]. Although HNF-4α was increased in STZ-diabetic rats, consistently with what has been reported by other authors [20], tungstate did not restore its levels. In the case of FoxO-1, neither diabetes nor the treatment with tungstate modified the expression of this factor (Figure 3B). However, we found a clear tungstate-induced decrease in the expression of peroxisome proliferator-activated receptor-gamma coactivator 1 alpha (PGC-1α) (Figure 3C), a cofactor required for the complete activation of PEPCK and G6Pase expression through specific interaction with HNF-4α and FoxO-1 [21].

### Cell-autonomous effects of tungstate on the regulation of gluconeogenesis

**Figure 4:**
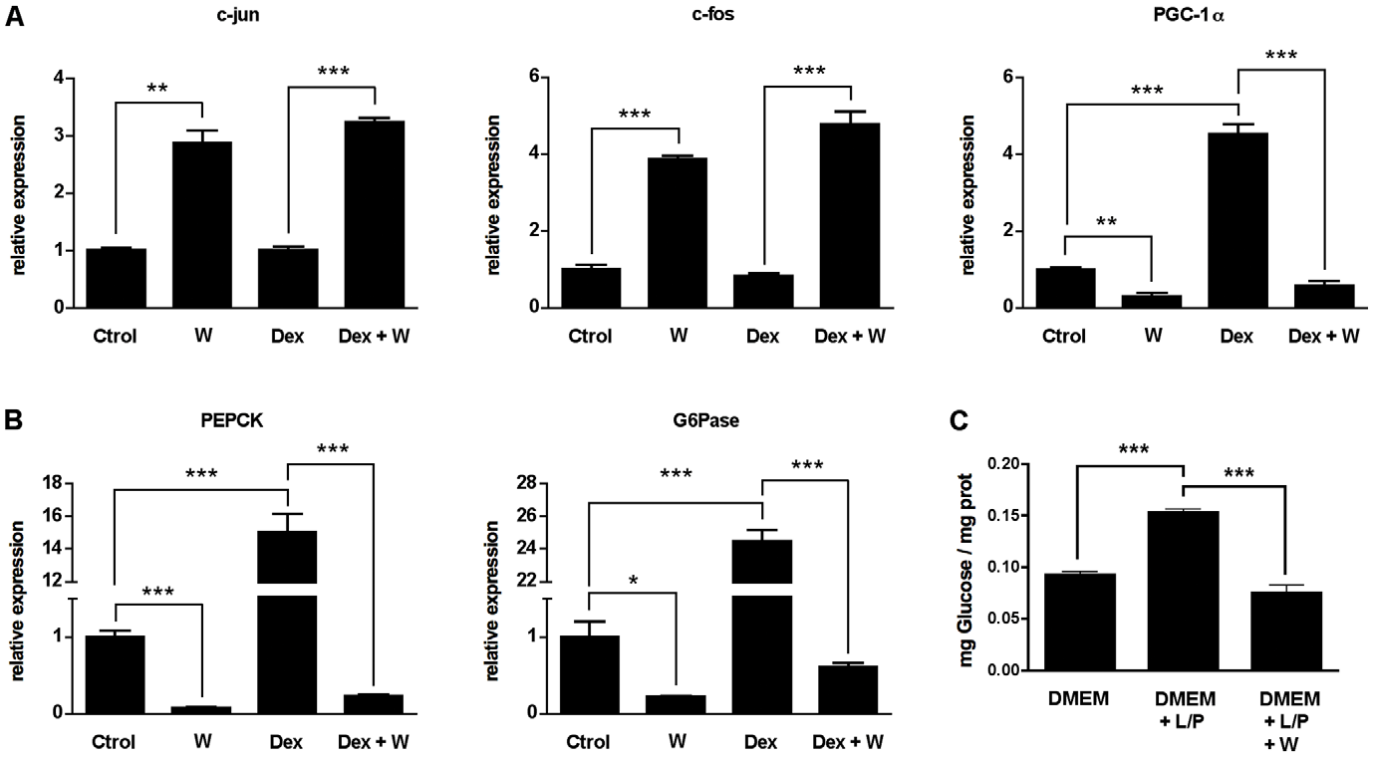
Inhibitory effects of tungstate on gluconeogenesis *in vitro*. Total mRNA was extracted from primary cultured rat hepatocytes treated with Dexamethasone 100 nM (Dex), sodium tungstate 1 mM (W) or both (Dex+W) for 18 h as indicated, and Real time-PCR was performed. Tungstate treatment increased the expression of c-jun and c-fos alone and in the presence of dexamethasone, whereas PGC-1α mRNA levels were decreased under these two conditions (**A**). Conversely, both PEPCK and G6Pase were downregulated after the treatment with tungstate alone (W) or in the presence of dexamethasone (Dex+W) (**B**). **C** Glucose output was measured in primary cultured hepatocytes incubated with the gluconeogenic substrates lactate (L) and pyruvate (P), as indicated. Data are representative of three experiments performed in triplicate. Error bars represent S.E.M. *, P<0.05; **, P<0.01; ***, *P<0.001*.

Tungstate treatment ameliorated hyperglycemia and improved the diabetic state by affecting hepatic metabolism and extra-hepatic targets. To differentiate the former from the latter, we performed experiments on primary cultured hepatocytes. In order to stimulate the gluconeogenic pathway, we treated rat primary cultured hepatocytes with tungstate in the absence or presence of dexamethasone [22], [23] for 18 h. We next analyzed changes in the expression pattern of the main gluconeogenic factors. Our data show that tungstate increased the expression of c-jun/c-fos and blocked the dexamethasone-induced expression of PGC-1α (Figure 4A). Dexamethasone-mediated induction of PEPCK and G6Pase expression was fully blocked by treatment with tungstate (Figure 4B). Noteworthy, all these experiments were performed in the absence of insulin and after 18-h glucose starvation. In order to confirm the effects of tungstate treatment on glucose output, primary rat hepatocytes were starved overnight and then cultured in medium supplemented with lactate and pyruvate to provide the substrates for gluconeogenesis. De novo glucose synthesis was blocked in tungstate-treated hepatocytes when compared with non-treated ones (Figure 4C).

## Discussion

**Figure 5:**
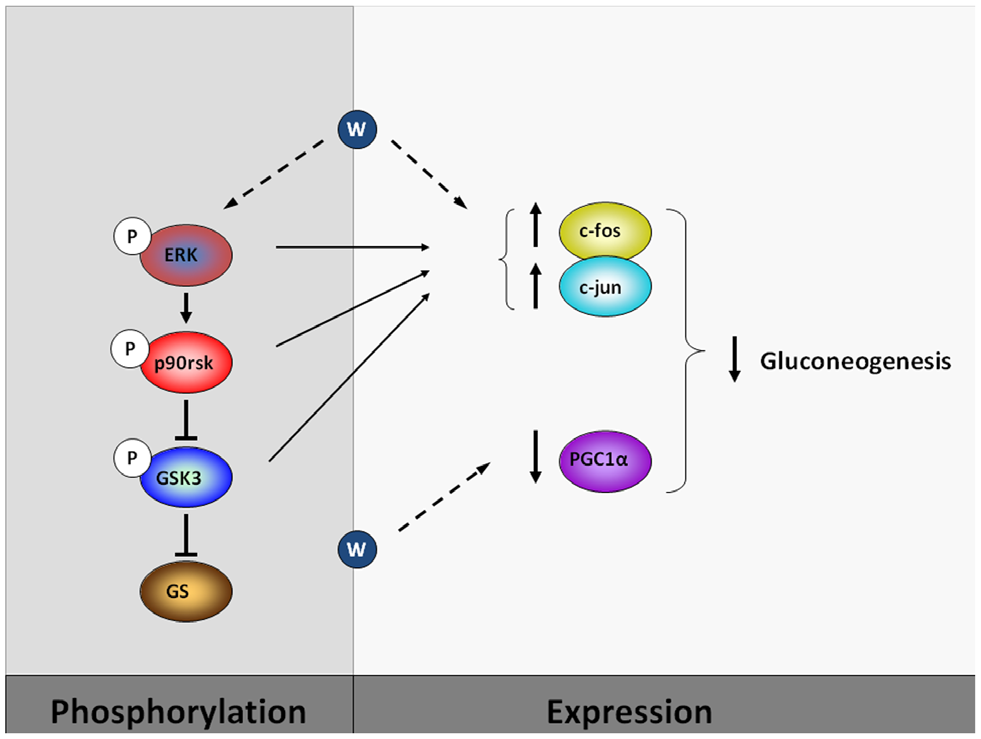
Schematic representation of the effects of tungstate. Treatment of diabetic rats and of cultured hepatocytes with tungstate (W) led to changes in the phosphorylation of signaling proteins and in the expression of transcription factors and gluconeogenic genes. All these changes caused a reduction in hepatic gluconeogenesis, thus contributing to the improvement of the diabetic state.

The liver is a key organ in the regulation of metabolism, especially glucose homeostasis, in which hepatic gluconeogenesis is crucial. The expression of PEPCK in liver is greatly increased in the diabetic state, thus leading to enhanced glucose production and contributing to hyperglycemia. It has been reported that tungstate increases hepatic glycolysis and glycogen synthesis while reducing gluconeogenesis [1], [24]. Here we report for the first time that tungstate restores the phosphorylation state of the MAPK ERK-1/2, GSK3 and p90rsk in livers of STZ rats and that this effect may explain how this compound modifies the expression of the transcription regulators c-jun, c-fos and PGC-1α, thus compromising the expression of gluconeogenic enzymes (Figure 5).

Several groups have described differences in the phosphorylation of ERK-1/2 in most tissues of STZ-induced diabetic rats. However, data are controversial, as increases and decreases have been reported in ERK phosphorylation in STZ models depending on tissue and time. For instance, ERK-1/2 phosphorylation is increased in the kidneys [25], [26] and also in the hearts [27] of rats 4 weeks after the treatment with STZ. In contrast, other authors report a decrease in ERK phosphorylation in heart and white gastrocnemius muscle in rats two weeks after STZ injection [28]. A report from 2001 also showed an increase in ERK phosphorylation in livers of ob/ob mice [29]. Nevertheless, until now changes in the phosphorylation state of ERK in the liver of STZ rats have not been reported. Here we show a clear decrease in ERK phosphorylation in the liver of this rat model and most interestingly a complete recovery after treatment with tungstate.

It has been proven that increased ERK activity leads to augmented p90rsk phosphorylation and consequently to that of GSK3 [30], [31], [32]. Inhibition of GSK3 by phosphorylation induces the activation of GS [33]. We have previously demonstrated that the *in vitro* activation of glycogen deposition in response to tungstate is due to GSK3 inhibition mediated by ERK phosphorylation [8]. Our group has also reported the activation of glycogen synthesis by tungstate *in vivo* [1], [2], [24], [34] and the results presented here support the notion that tungstate induces glycogen deposition *in vivo* by the same mechanism.

Furthermore, ERK-1/2 activate c-fos through its phosphorylation as well as by increasing its expression [15], [35]. c-jun and c-fos are components of the AP1 complex, which decreases the expression of gluconeogenic genes, such as PEPCK [16]. Interestingly, GSK3*β* inactivation leads to enhanced cellular c-jun levels [36]. Thus, the inhibitory effect of tungstate on GSK3 could contribute to AP1 activation by increasing c-jun expression. Moreover, p90rsk has been described not only to inactivate GSK3 [30], [31], [32] but also to directly trigger the activating phosphorylation of c-fos [37], [38], [39], thereby resulting in a decrease in gluconeogenesis. The transcriptional regulation of metabolic genes is a complex process involving the expression and activation of several transcription factors. In addition to AP1, it has been reported that FoxO-1 and HNF-4α transcriptionally control a number of metabolic genes involved in gluconeogenesis [17], [18], [19]. FoxO-1 is also involved in the regulation of glycolysis [40]. Moreover, the specific interaction of PGC-1α with FoxO-1 and HNF-4α is known to be required for the complete activation of PEPCK and G6Pase expression [21]. Our data indicate that while FoxO-1 expression is not altered in the liver of diabetic rats, HNF-4α and PGC-1α are strongly upregulated. Tungstate treatment does not affect the expression of HNF-4α but represses that of PGC-1α in diabetic livers, thus further compromising the expression of gluconeogenic genes.

Tungstate treatment reduced water intake in both diabetic and healthy rats. The drastic reduction in water intake of treated diabetic animals can be attributed to the normalization of glycemia, since it occurred in parallel to the decrease in blood glucose levels. However, treatment of healthy animals with tungstate had no effect on the parameters measured, indicating that the effects observed *in vivo* are not a result of altered water intake. We observed moderately increased insulinemia in tungstate-treated STZ-diabetic rats, possibly as a result of the reported tungstate-mediated regeneration of pancreatic beta-cell population [41]. However, basal insulinemia in treated diabetic rats was still half that registered in healthy rats. While this increased insulin production could contribute to reduce glycemia by both intrahepatic and peripheral actions, the lack of changes in the phosphorylation state of hepatic PKB/Akt of these animals indicates that the effects of tungstate on the liver are insulin-independent. The phosphorylation of PKB/Akt is a canonical effect of insulin [42] that occurs through the activation of PI3K and PDK1 [43], [44] and in all tissues responsive to insulin, such as the liver. PKB/Akt has been proposed to mediate insulin-induced reduction of hepatic gluconeogenesis by several mechanisms [45]. In addition, the notion that the actions of tungstate on the liver are insulin-independent is also supported by our observations that the effects of this compound on gluconeogenic enzymes are maintained in isolated hepatocytes cultured in the absence of insulin.

Taken together, our findings indicate that the antidiabetic agent sodium tungstate restores intrahepatic ERK phosphorylation, and the downstream signaling and expression of transcription factors, which in turn may regulate the expression of several metabolic genes in a rat model of diabetes mellitus. Our results indicate that tungstate modulates hepatic glucose production, one of the most relevant processes in the regulation of glucose homeostasis and one that is altered in diabetes. These actions appear to be independent of insulin signaling, as shown in an *in vitro* model of induced gluconeogenesis, and may contribute to ameliorating hyperglycemia. By studying the mechanism of action of tungstate, we have pinpointed key therapeutic targets that control glucose homeostasis. These targets deserve further exploration in the search for more efficient treatments for diabetes.

## Supporting Information

Figure S1 **Water intake for each experimental group during the 30-day treatment.** Treated animals were given tungstate dissolved in distilled water, whereas non-treated animals were given tap water. Only diabetic and diabetic-treated animals were caged individually. Water intake was measured every 3 days for each cage. Measurements are expressed as the mean of ml/day per animal. Error bars represent S.E.M.
(TIF)
