# Supplementary figures and images for: Tungstate Reduces the Expression of Gluconeogenic Enzymes in STZ Rats
Source: PLoS One. 2012 Aug 8;7(8):e42305. doi: 10.1371/journal.pone.0042305 (PMC3414523; doi:10.1371/journal.pone.0042305)

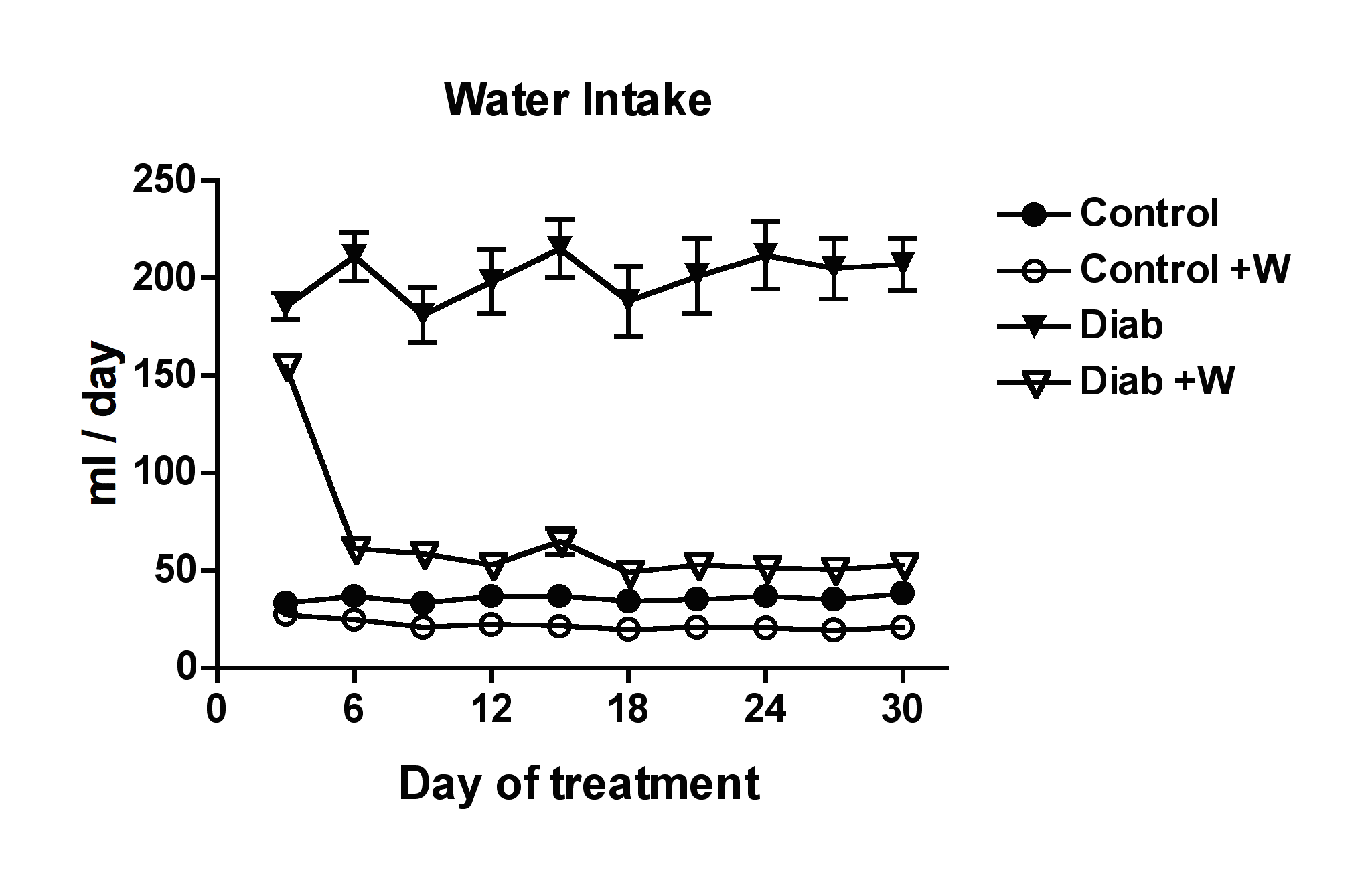

Supplement: Figure S1 — Water intake for each experimental group during the 30-day treatment. Treated animals were given tungstate dissolved in distilled water, whereas non-treated animals were given tap water. Only diabetic and diabetic-treated animals were caged individually. Water intake was measured every 3 days for each cage. Measurements are expressed as the mean of ml/day per animal. Error bars represent S.E.M. (TIF) [file pone.0042305.s001.tif]
